# Young adult males’ motivators and perceived barriers towards eating healthily and being active: a qualitative study

**DOI:** 10.1186/s12966-015-0257-6

**Published:** 2015-07-15

**Authors:** Lee M Ashton, Melinda J Hutchesson, Megan E Rollo, Philip J Morgan, Debbe I Thompson, Clare E Collins

**Affiliations:** School of Health Sciences, Faculty of Health and Medicine, Priority Research Centre in Physical Activity and Nutrition, University of Newcastle, Callaghan, Australia; School of Education, Faculty of Education and Arts, Priority Research Centre in Physical Activity and Nutrition, University of Newcastle, Callaghan, Australia; USDA/ARS Children’s Nutrition Research Center, Department of Pediatrics, Baylor College of Medicine, Houston, USA

**Keywords:** Young men, Focus groups, Qualitative research, Physical activity, Healthy eating, Obesity, Health behaviour

## Abstract

**Background:**

There is a lack of understanding of young men’s perspectives in obesity-related research. This study aims to: (1) identify young men’s perceived motivators and barriers in adopting healthy eating and physical activity behaviours, and (2) explore any differences in responses by weight status categories.

**Methods:**

Ten focus groups (32-63 minutes; 3-9 participants per group) were conducted with 61 young men (BMI: 25.3 ± 5.1 kg/m^2^, aged: 18-25 years) from the Hunter region, New South Wales, Australia. There were 35 (57.4 %) healthy weight men and 26 (42.6 %) overweight/ obese men. Three groups were with healthy weight participants, three with overweight/obese participants and four with mixed-BMI participants. Sessions were audio-recorded and transcribed. Data analysis was conducted by an independent researcher using NVIVO10.

**Results:**

Motivators for healthy eating grouped into four themes: physical health (e.g. to live longer), sport or performance (e.g. to support their sporting goals), physical appearance (e.g. sexual attractiveness) and social influences (e.g. societal expectations to eat healthy), while key motivators for physical activity were: physical appearance (e.g. sexual attractiveness), social inclusion (e.g. making friends), physical and mental health (e.g. relieve stress) and improvements for sport or performance (e.g. improve fitness). Themes for key barriers to eating healthy were: intrinsic (e.g. perceived effort to adopt healthy eating), logistic (e.g. cost), and social factors (e.g. peer influence), while busy lifestyles (e.g. lack of time), logistic (e.g. cost), cognitive-emotional (e.g. feelings of inferiority) and social factors (e.g. family upbringing) were key barriers for physical activity. Responses varied little by BMI status.

**Conclusion:**

This research emphasises the importance of consulting young men when developing healthy lifestyle programs that aim to promote healthy eating and physical activity in young men. Future research is needed to identify the most effective ways to address their motivators and barriers in intervention research.

## Background

Young adult men (aged 18-25 years) are entering a key transitional life phase. During this time period many personal, social and environmental changes transpire such as moving away from home [1], starting tertiary education [1], cohabitation with peers or partners [1], and beginning employment [2]. These changes can be associated with unhealthy lifestyle behaviours such as weight gain [3], poor dietary behaviours [4, 5] and sedentary lifestyles [6, 7].

Globally, young men have a higher prevalence of overweight and obesity compared to young women (~18 % vs ~17 %) and these differences are more pronounced in developed countries (~37 % vs ~29 %) [8]. This is mirrored in Australia (42 % vs 35 %) [9], which is not surprising given that almost half (48 %) of young Australian men fail to meet national physical activity recommendations and approximately 97 % fail to consume adequate intakes of fruit and vegetables daily [9]. Of particular concern is the steep trajectory of weight gain starting at 18 years, such that by age 35-39 years nearly 60 % of males from developed countries are classed as overweight or obese [8]. Once established obesity is hard to treat; a recent systematic review confirmed only 14 out of 56 interventions achieved significant results for weight loss maintenance (>5 % of initial weight loss) [10]. Therefore prevention of obesity is critical.

A Danish study in young men found that those who were obese at age 20 years were twice as likely to die by age 55 years than their non-obese counterparts and had an 8-year shorter life expectancy [11]. This highlights the critical importance of addressing key obesity prevention lifestyle behaviours in men whilst they are still young.

Programs have been implemented that target health risk behaviours (e.g. diet and physical activity) in young men, but many utilize a ‘one-size fits all’ approach [12]. This is problematic because males and females differ, as they are likely to have unique perceived motivators and barriers to modifying their lifestyle behaviours [13]. The limited knowledge of young men’s perspectives in obesity-related research has been gathered from studies in university/college students with samples overrepresented by women [14, 15].

In a recent systematic review of health-related interventions that recruited young men only (Ashton *et al* ‘unpublished observations’), none reported using a participatory approach, indicating that the interventions were designed without taking into account young men’s perceived motivators and barriers. Interventions designed without the perspective of the target audience are at risk of having decreased engagement, adherence and impact [16]. The value of a participatory approach is its potential to bridge gaps between research and practice by identifying key motivators and barriers to healthy lifestyle choices, ultimately enabling the design of a more tailored intervention [17].

There is a general lack of understanding as to why young men do not engage in health interventions and how to design a program that appeals to them. Few studies have addressed this. A qualitative study in the US explored college students’ barriers to weight management [18] and split the sessions by sex. The focus group sessions containing only young men identified intrapersonal (e.g. lack of discipline), interpersonal (e.g. social situations) and environmental (e.g. time constraints) barriers. Similarly, another qualitative study in the US [19] explored both motivators and barriers to healthy weight maintenance in young male college students. The barriers identified were comparable to Greaney *et al* [18], but additional barriers included lifestyle obligations, influence of female partners on sedentary behaviours and disliking the taste of healthy foods. Motivators were generally centred on appearance (e.g. to be more attractive), addressing medical issues (e.g. long term health) and achieving sporting goals [19]. However, both of these studies were conducted with university student populations from the US, and thus may not provide a broad representation of motivators and barriers. A recent focus group study in rural, Australian, [20] overweight and obese young men recruited from various community settings aimed to explore attitudes towards losing weight. Although the strict population specifications resulted in a small sample size (n = 30), valuable information was obtained. The young men said being overweight or obese was the ‘norm’ in today’s society and thus did not feel the need to change. Environmental factors (e.g. price and convenience of fast food) were identified as key barriers to changing weight status.

This current study aims to build on this limited evidence base by reporting results of focus groups conducted in a sample of young men aged 18 to 25 to: 1) identify perceived motivators and barriers in adopting healthy eating and physical activity behaviours and 2) explore any differences in responses by weight status categories.

## Methods

The conduct and reporting of this paper adhered to the guidelines outlined in the consolidated criteria for reporting qualitative research (COREQ) [21]. Ethical approval was obtained from the University of Newcastle Human Research Ethics Committee.

### Study design

A qualitative design using a focus group methodology was applied as they are an ideal way to explore young men’s perceived motivators and barriers to healthy behaviours. This approach was selected because focus groups can be undertaken in naturalistic settings as opposed to experimental situations [22] which may stimulate more openness and candour [23]. Also the group interaction has the capability to elicit information and insights that are less accessible during individual interviews [24]. Probing by the moderator allowed in depth exploration of unanticipated issues as well as an opportunity to clarify and enhance understanding of responses [23].

### Participants and setting

Eligible participants included: male and aged between 18 and 25 years who were fluent in reading, writing and speaking in English. Young men were selected using a purposive sampling method (i.e. homogenous on a particular characteristic; in this case it was for gender and age) and recruited from the local university, technical colleges (TAFE) and the community. Recruitment materials included: flyers distributed around the university and technical college campuses, a media release (newspaper, local radio and social media) and advertising on social media (Facebook). Interviews continued until the moderator felt that data saturation was achieved and this was confirmed during data analysis. Interested individuals were asked to complete an online eligibility screen. Eligible individuals were contacted via email. Demographic characteristics were collected during the online eligibility screen including self-reported height and weight which were used to calculate BMI. Consented participants were allocated to a focus group session based on BMI status; three groups consisted of healthy weight males (BMI: 18.5-24.9 kg/m^2^), three were with overweight/obese participants (BMI: ≥25.0 kg/m^2^), while the remaining four groups consisted of mixed-BMI participants. Focus groups were conducted in a private venue at the university or technical college. Participants received a $25 gift voucher to cover time and travel costs.

### Data collection

Each focus group contained between 3-9 participants, with focus groups lasting between 32 to 63 minutes. All groups were conducted by the first author (LMA) who at the time of the study was a PhD student and classed as a young male (aged 25 years). The interviewer received detailed training in focus group methodology by a highly experienced practitioner (DIT). An assistant moderator (male, aged 27 years) attended all sessions to aid with note-taking and time management. No relationship between the interviewer and participants was established prior to study. All participants provided written informed consent.

The structured discussion framework was developed by the research team to facilitate discussion and reflection around the overall aims of the qualitative study. The questions were generated using the Krueger and Casey’s method for ‘five categories of questions’ [25] which include an opening question, introductory question, transition questions, key questions and a concluding question. After review of the literature, the content of questions was informed by the questions from similar studies in young men [18, 19]; these were then pretested with small groups of young men and revisions made where necessary. A total of eleven questions were asked but only the responses to four questions are included in this paper. Specifically the included questions explored participants’ motivators and barriers to healthy eating and physical activity. Probes were used to clarify, and explore the topics. In the closing minutes of each focus group session a verbal summary of responses was provided to participants by the moderator. Participants were asked to review the summary and then had the option to provide any other additional comments that may have been missed. Once the participants had departed, both the moderator and assistant moderator had a brief discussion to compare notes and ensure the perceptions of responses were the same.

### Data analysis

The focus groups were digitally recorded with the participants’ consent and transcribed verbatim. A computer program (NVIVO 10) was used to assist with the organisational aspects of data analysis. Analysis was conducted by an independent qualitative researcher. A hybrid approach of inductive and deductive analysis was adopted [26] allowing for an in-depth exploration of data-driven, as well as theory driven concepts. A coding template was developed a-priori based on the research questions which was then applied to the data with the intent of identifying meaningful units of text. The coding scheme was revised and further expanded during coding, as new inductive codes were assigned to segments of data describing a new theme. Following coding of all focus group transcripts, themes and patterns in the data were identified and connected into an explanatory framework, clustered under headings that directly related to the research questions. This interpretative phase enabled the generation of overarching and sub-themes which were felt to capture the multifaceted views, experiences and insights of participants. All themes are presented in weighted order with the most frequently mentioned first.

## Results

Sixty one young men (mean age: 20.8 ± 2.3 years) from the Hunter region, New South Wales, Australia participated in 10 focus groups (average 6.1 participants per group). Of these 35 (57.4 %) were healthy weight and 26 (42.6 %) were either overweight or obese. Participants were predominantly single (91.8 %) and approximately half were university students (52.5 %) (Table 1).

**Table 1:** Demographic characteristics of young adult men (n = 61)

Characteristic	Mean ± SD or n (%)
Age (years)	20.8 ± 2.3
*Employment status*	
Student (University)	32 (52.5 %)
Student (technical college)	14 (22.9 %)
Working full time paid employment	10 (16.4 %)
Casual employment	3 (4.9 %)
Unemployed	2 (3.3 %)
*Highest Qualification*	
School certificate (Year 10 or equivalent)	3 (4.9 %)
Higher school certificate (Year 12 or equivalent)	40 (65.6 %)
Trade/ apprenticeships	1 (1.6 %)
Certificate/ diploma (e.g. childcare, technician)	8 (13.1 %)
University degree	9 (14.8 %)
*Income*	
No income	13 (21.3 %)
$1 - $199 per week, ($1 - $10,399 per year)	6 (9.8 %)
$200 - $299 per week, ($10,400 - $15,599 per year)	16 (26.2 %)
$300 - $399 per week, ($15,600 - $20,799 per year)	3 (4.9 %)
$400 - $599 per week, ($20,800 - $31,199 per year)	2 (3.3 %)
$600 - $799 per week, ($31,200 - $41,599 per year)	1 (1.6 %)
$800 - $999 per week, ($41,600 - $51,999 per year)	2 (3.3 %)
$1,000 - $1,249 per week, ($52,000 - $64,999 per year)	3 (4.9 %)
$1,250 - $1,499 per week, ($65,000 - $77,999 per year)	2 (3.3 %)
$1,500 - $1,999 per week, ($78,000 - $103,999 per year)	1 (1.6 %)
Did not Know	10 (16.4 %)
Did not want to answer	2 (3.3 %)
*Marital status*	
Single	56 (91.8 %)
Defacto	4 (6.6 %)
Married	1 (1.6 %)
Height (m)	1.8 ± 0.6
Weight (kg)	81.1 ± 16.7
Body mass index (kg/m^2^)	25.3 ± 5.1
*BMI category*	
Healthy weight	35 (57.4 %)
Overweight	17 (27.9 %)
Obese	9 (14.7 %)

### Factors motivating young men to engage in healthy eating

Young men were asked; “*Please tell me a few reasons why young men like you might want to eat healthier foods?”* Four themes emerged: 1) physical health; 2) sport or performance; 3) physical appearance and 4) social influences. The themes did not differ by weight status and are therefore reported combined.

#### Physical health

Most participants mentioned health or medical benefits as a reason for eating healthier foods. This included both short-term benefits (e.g., general body functioning and immune system) and long-term benefits (e.g., increasing life expectancy). As one participant said: *“Well I guess when you eat healthier you…well your body functions better and you feel better…” (overweight/ obese).* Another said: “*longevity too so like living longer, not getting sick as often” (healthy weight).*

#### Sport or performance

Young men were also motivated to eat healthier to complement athletic/sporting goals or other lifestyle pursuits. As one participant stated: *“Oh yes to support their goals in the gym or in sport is the primary reason for a lot of my mates” (overweight/obese)* while another said: *“To perform better like at sport and like at work” (overweight/obese).*

#### Physical appearance

Many young men acknowledged the positive impact healthy eating had on improving their physical appearance and attractiveness to potential partners. In particular the potential to lose weight and be more appealing and attractive to others seemed to be especially important. For instance one young man said: *“To look good and attract the women” (overweight/obese).*

#### Social influences

Social influences on healthier eating were divided into two sub-themes. Social inclusion emerged, with many young men discussing the positive expectations attached to healthy eating: *“I just kind of feel like it’s what’s you’re supposed to do, like it’s just that thing you know you’re supposed to be doing when it comes to food” (overweight/obese).* Also social environment, including peers and family were influential on food choices: “*Yeah like if your parents are health nuts whatever then you’ll grow up as wanting that stuff” (overweight/obese).*

#### Factors motivating young men to undertake physical activity

Participants were asked: “*Please tell me a few reasons why young men like you might want to be more physically active?”* Responses were divided into four themes: 1) physical appearance; 2) social inclusion; 3) physical and mental health and 4) sport or performance. Each is discussed below. Similar to healthy eating, themes did not differ by BMI status.

#### Physical appearance

Amongst the most frequently mentioned motivators were factors relating to body image, such as “*getting a hot body” (overweight/obese)* and ultimately becoming more attractive to others, particularly the opposite sex: *“I think a lot of people are motivated by aesthetics and looking good with the hope to attract a partner probably” (overweight/obese).*

#### Social inclusion

Physical activity was viewed by many as providing an important context for gaining social acceptance, making friends and improving one's standing amongst peers (i.e., impressing others and gaining popularity). As one participant said: *“The social aspect of getting involved in a sporting club and spending time with your mates, all that sort of stuff” (overweight/obese)*

#### Physical and mental health

The motivators to undertaking physical activity were similar to the motivators identified for healthy eating, with frequent reference to improving overall physical health and the effect this may have on longevity. However the motivators to physical activity included more emphasis on the mental health benefits associated with physical activity, particularly by its stress relieving and ‘feel-good’ potential through the effect of endorphins and adrenaline, or simply through the pure enjoyment of physical activity. For instance one participant reported: *“Feel better like endorphins and all that razzmatazz” (healthy weight).*

#### Sport or performance

Many young men were motivated by the prospect of developing new sport skills: with common examples including: “*to build on strength*” *(healthy weight)* and “*to improve fitness” (overweight/obese).* They were also motivated by the sense of achievement associated with mastery of the new physical skill: *“…and there’s something motivational about learning a new sport as well or a new you know like surfing, skateboarding whatever if you’re not good at it and you start mastering it, it’s kind of motivational to keep going with it” (healthy weight).*

### Barriers to healthy eating

Participants were asked: “*Please tell me a few reasons why young men might not eat healthy already?”* All groups raised issues that broadly fell into three themes: 1) factors intrinsic to the person; 2) logistic factors and 3) social factors.

#### Factors intrinsic to the person

Amongst the intrinsic barriers mentioned, the perceived effort required to adopt healthy eating patterns was amongst the most commonly mentioned. Healthy foods were perceived to require a greater degree of planning and preparation; as one young man said: “*It certainly takes much more time to think about preparing a balanced diet for a week and hitting everything, all the food groups and a variety of foods and preparing that all and stuff which, I guess if you are busy, you’re not going to prioritise it, there’s always something else more urgent” (overweight/obese).*

There were differences in responses for some of the intrinsic factors by BMI status; only healthy weight young men reported lack of knowledge and lack of skills required in relation to buying, preparing and cooking healthy foods. As one participant said: “*I think another big one is not knowing how to prepare such foods, I think there’s lots of various healthy foods that you just don’t know what to do with them most of the time” (healthy weight).* Although this barrier was mentioned by healthy weight men, it did not appear to be specific to them i.e., it appeared that their perception was that this was thought to be a general barrier faced by all young men when attempting to eat healthy, regardless of weight status.

#### Logistic factors

Participants in most focus groups identified access and cost as key logistic barriers. Overall, unhealthier foods were perceived by the majority to incur lesser costs with one participant providing the example of the ubiquitous “*$2 McDonalds burger” (overweight/obese)* and as being omnipresent in society: *“in terms of fast foods like you can quickly get them and they’re easy to obtain generally” (overweight/obese).*

#### Social factors

Many young men identified important social barriers to adopting healthy eating. The most commonly mentioned were the social context within which most eating occurred, emphasising the role that peers especially had on the choice of foods consumed; as one participant reported: *“I think depending on like the situation or whatever, quite a lot of like what we would be doing is eating, is more of a social type thing so when we are eating, we’re eating in a group and it’s not like “Hey, come over, let’s all have a salad together” (overweight/obese).* In addition many young men acknowledged the societal expectations placed upon them which did not support a healthy diet such as male stereotypes and the stigma attached to eating healthy foods, as one young man said: “*It [healthy eating] goes against the sort of stereotypical masculine image in society” (overweight/obese).*

### Barriers to physical activity

Participants were asked: “*Please tell me a few reasons why young men might not be physically active already?”* four themes emerged: 1) busy lifestyles; 2) logistic factors; 3) cognitive-emotional factors and 4) social factors. Participants listed similar physical activity barriers regardless of BMI status, although there was a trend in the data for participants in the healthy weight groups to discuss barriers in a more hypothetical manner, while those in the overweight/obese groups often discussed barriers in a more personal context.

#### Busy lifestyles

Almost all participants mentioned busy lifestyles as a key barrier; in this context lack of time, lack of motivation, lack of commitment and influence of other competing leisure activities were relayed as very prominent obstacles contributing to a busy lifestyle. As a result young men often prioritised other activities/commitments ahead of physical activity: *“I’d say lack of time for me, I have a lot of other things to do and I kind of tend to push physical activity like right down to the bottom of the list”(healthy weight).*

#### Logistic factors

As was the case for barriers to healthy eating; almost all participants mentioned cost and access as key barriers to physical activity. Particularly access to a variety of sporting activities and the costs associated with gym membership and equipment, as one young man said: *“I didn’t join the University Tennis Club for first year because I just didn’t have the money for a hundred a semester” (overweight/obese).*

#### Cognitive-emotional factors

Many young men often felt that doing exercise or going to the gym was associated with feelings of inferiority, inadequacy, lack of self-confidence and feeling self-conscious or embarrassed. Negative stereotypes and the stigma attached to the gym activities were also frequently mentioned: “*I just hate the idea of firstly people yelling at me and them people with Spandex” (overweight/obese).*

Many participants also talked of having unrealistic goals. Not being able to achieve such goals or finding that one is not as good at an activity as expected was seen as a deterrent to future physical activity. As one participant responded: *“Sometimes you try to be physically active and then you’re not quite that good and so you…like you try and overdo it at first and then you kind of regress and just go back to doing nothing because you couldn’t do everything”(healthy weight).*

#### Social factors

Many young men identified the influence of peers, social group membership (i.e. university student) and family upbringing as barriers to physical activity. As one participant said: *“if your friends don’t work out and go to the gym, it’s unlikely that you’ll go” (overweight/obese)* and another said: *“Past times, just how you grew up, if you grew up watching TV well you’re going to watch TV” (overweight/obese).*

## Discussion

The aims of this qualitative study were to provide an in-depth insight into young men’s facilitators and barriers to healthy eating and physical activity and to explore any differences in responses by weight status category. The dominant motivating themes to eating healthier foods included; improving physical health, complementing sport or performance, improving physical appearance, and social influences (e.g. positive family influence). Similar motivating themes were apparent for undertaking physical activity including; improving physical appearance, social inclusion, improving physical and mental health, and improving sport related skills or performance.

The leading barriers to healthy eating as identified by young men include: factors intrinsic to the person, logistic (e.g. cost and access) and social factors. Perceived barriers to physical activity include: busy lifestyles (e.g. lack of time), logistic (e.g. cost and access), cognitive-emotional and social factors. There were minimal differences in the responses for all questions between young men classified as healthy weight and those classed as overweight/obese.

The current findings are mostly consistent with Walsh *et al* [19] where young male college students reported similar physical health benefits (e.g. long-term health), complementing sporting performance and physical appearance benefits (e.g. sexual attractiveness) as key motivating themes to healthy eating. This is also apparent for physical activity motivators with similar themes identified, such as improvements in physical health (e.g. current/future health), mental health (e.g. to improve state of mind), sporting skills (e.g. fitness) and physical appearance (e.g. attractiveness). The high proportion of single young men in the Walsh *et al* [19] study (87 %) is comparable to the current study (92 %) and may explain why both identified attractiveness to future partners as a key theme. However unlike the current study, no social motivating themes arose for either physical activity or healthy eating in their analysis. This needs be explored further to determine whether social inclusion and positive influences of peers and family can motivate young men to improve health behaviours.

The perceived barriers influencing healthy eating and physical activity in the current study support earlier research that lifestyle factors (e.g. time constraints [18, 19] and other obligations, such as job and education schedule [19]), social factors (e.g. peer influence [20]) and logistic factors (e.g. lack of access to healthy foods and high costs associated with healthy behaviours [18, 20]) are major influences. Whilst current findings confirm these key barriers, additional social barriers (e.g. stereotypes placed upon young men), cognitive-emotional barriers (e.g. feelings of inferiority when doing physical activity) and intrinsic barriers (e.g. perceived effort to adopt healthy eating) were also identified. The additional responses in the current study may be due to the larger sample or the inclusion of young men with different socio-demographic characteristics. Of the three other studies, two were conducted in university/college students from the US [18, 19] and one in rural Australian young men [20] whereas the current study includes young men from a range of socio-demographic backgrounds; for example, although 75 % of the current sample were students, 23 % were from technical colleges whereby a distinguishing feature is an over-representation of students from low socio-economic areas [27]. This is also reflected by the low average income rates of the young men (between $14,963- $21,170 per annum) when compared to the national average income for young Australian men aged 15-24 years ($27,671 per annum) [28].

Notably, young men in the current study identified social factors as both motivators (e.g. social inclusion) and as barriers (e.g. peer influence) to healthy eating and undertaking physical activity. Similarly Mendis *et al.* [20] found young men reported that social activities with peers can impact negatively on food choices but also found that social support is required to motivate them into making positive lifestyle behaviour changes. The positive and negative aspects of social influences must be considered in future health-related interventions with young men, with attempts made to specifically focus on the positive aspects such as social support and social inclusion. At the same time, counteracting the negative aspects of peer influence (e.g. on unhealthy eating habits) is imperative. A potential way of addressing this would be to target the social group; results of meta-analytic reviews confirmed individuals were more likely to adhere to exercise programs if they participated with others in social or group-based settings rather than on their own [29, 30] and increased total energy expenditure when supported by friends or family [31].

Surprisingly, there were very few differences in the responses by weight status. This suggests that there are either no differences in young male’s perspectives by BMI status, or could potentially be due to inaccurate self-reporting of weight status [32] and thus some participants could have been stratified into the wrong group. However a recent study has demonstrated the validity of online self-report of height and weight by young adults [33]. In addition, BMI does not reflect muscle mass content [34], young men with a greater muscle mass may have been stratified into the overweight/obese group and the responses by these individuals may have suppressed the comments from young men who have a higher fat mass. Also several overweight or obese young men only wanted to attend sessions with their healthy weight friends (hence the mixed-BMI group sessions). This may have resulted in some overweight/obese young men mirroring responses to appease or agree with their healthy weight friends out of a desire to “fit in”.

The European Commission on Men’s Health recently highlighted that the behavioural differences between men and women represent different needs and perceived barriers in terms of health promotion and intervention design [13]. To some extent this is supported in the responses from the current study as motivators (e.g. to develop sporting skills) and barriers (e.g. male stereotypes and stigma attached to healthy foods) unique to young men were identified, that were not identified in online surveys of young Australian women [35, 36]. Whilst there are similarities for motivators (e.g. to improve physical and mental health) and barriers (e.g. time constraints and cost) to weight change in young women these may be due to age related factors as both sexes are experiencing similar transitional phases in their life such as; starting tertiary education [1, 37] cohabitation with peers or partners [1] and less financial stability (compared to older adults) particularly for Australian young adults where full-time employment rates are at their lowest since records began in 1986 [37]. The age-related differences are apparent when compared to similar studies with mostly middle-aged and older men [38–41]. Unlike the responses from this current study, improving appearance and sport skills (e.g. fitness) were perceived as less important motivators to exercise in older men (aged 40-55 years) [38]. Although there were some similarities such as improving health [38], older age groups of men were mainly motivated to exercise and lose weight to become more effective in their work role [38, 40]. In contrast to the responses from young men in the current study, negative influences from peers and logistic factors (e.g. cost) were not seen as barriers in older age groups of men [39–41]. Lifestyle differences between the age groups (e.g. occupational status, housing environment, family circumstances, marital status) may be attributable to these differences in barriers [42].

Early research has linked masculinities with young men’s health, often associating hegemonic masculinity with poorer health behaviours [43, 44]. Hegemonic masculinity is not a fixed entity but is often used to describe men who align with masculine ideals (e.g. strength, aggression, courage, independence, physical risk and being emotionally strong) [45, 46]. This hegemonic ‘macho’ masculinity was exhibited by several young men in the current study; for example, one young man said: “*It [healthy eating] goes against the sort of stereotypical masculine image in society” (overweight/obese).* Whilst another nonchalantly discussed many of the risk behaviours that they undertook: *“…and if you’re having more alcohol I’m going to have some drugs” (overweight/obese).* Recent research on masculinities have confirmed that young men are now constructing post-modern masculine identities because of changes to youth culture, leisure patterns and employment and there is a move away from the conventional hegemonic masculinity to a more pluralistic interpretation [2]. Gender tailored interventions (i.e. those designed to address the individual characteristics of persons within a sample, such as personality factors, goals, needs, preferences, and resources [47]) have the potential to address the social diversity and plurality of masculinities in young men [48]. Previous research has shown gender tailoring to be effective in improving health outcomes and recruiting, retaining and engaging men [49–51]. However, these studies were generally conducted in middle-aged and older men and therefore there is a need to test this in young men [12].

### Implications for research and practice

Healthy lifestyle programs that incorporate nutrition and physical activity for young men should be designed to address their motivators and offset their perceived barriers to facilitate behaviour change. Table 2 provides a list of potential program components, program messaging and recruitment strategies for a tailored program for young males to address the key themes emanating from the current study.

**Table 2:** Potential strategies to inform development of programs aiming to address motivator and barrier themes to healthy eating and physical activity

Theme	Potential strategies to address each theme
*Motivators for healthy eating*	
Physical Health	•Emphasise both proximal (e.g. improved energy levels) and distal health benefits (e.g. prevention of chronic diseases) of healthy eating in program messaging and recruitment strategies.
Sport or performance	•Nutrition educators to inform of ways in which certain foods and healthy eating can improve sporting performance and work related activity.
	•Provide daily meal plans of their sporting role models and how this contributes to different areas of performance (e.g. healthy foods to consume pre-workout or healthy foods to help with recovery)
Physical appearance	•Recruitment strategies and messaging to focus on ways the program can improve appearance e.g. to improve muscle mass.
Social influences	•Target social groups/circles and have group-based sessions
	•Tailor recruitment strategies to social groups e.g. “bring-a-mate”
*Motivators for physical activity*	
Physical appearance	•Recruitment strategies to focus on ways program can improve appearance e.g. improve muscle mass
Social inclusion	•Target social groups/ circles and have group-based sessions
Physical and mental health	•Emphasise both proximal and distal benefits of physical activity in program messaging and recruitment strategies.
	•Recruitment strategies and program messaging to focus on mental health benefits of physical activity e.g. decrease stress and ‘feel better’
Sport or performance	•Self-monitoring tools to determine progress of sporting skills (e.g. fitness and fat free mass)
	•Include a mixture of different sports to enable mastery of many different physical skills
*Barriers to healthy eating*	
Intrinsic	•Nutrition educators to offer guidance to cook quick, easy and nutritious recipes.
Logistic	•Nutrition educators to offer guidance for eating healthy on a budget
	•Intervention facilitators and young men to work together to make meaningful changes to the environment (e.g. targeting young male households)
	•Educate young men on how and where to access healthy foods (e.g. supermarket tour)
Social factors	•De-emphasise the masculine stereotypes e.g. focus on male role models such as sporting idols who eat healthily
*Barriers to physical activity*	
Busy lifestyles	•Flexibility in intervention delivery mode: for example, intervention sessions could be face-to-face but also video recorded to allow young men to attend in-person or relay the video recording at a time most convenient for them
Logistic	•Increased access to fitness facilities (e.g. improve knowledge of outdoor workout equipment ) or home-based fitness equipment
	•Promote exercise that does not need equipment
Cognitive-emotional	•Intervention facilitators to provide young men with encouragement and positive reinforcement.
Social factors	•Target social groups/ circles and have group-based sessions

### Strengths and limitations

The strengths of this study include; (1) a relatively large and diverse sample (n = 61) when compared to other similar studies in young men [18–20] and (2) the segmenting of focus groups by BMI status.

Limitations of the current study include; (1) the lack of diversity of the sample for employment status, with an overpopulation of students and (2) even though lower income rates were apparent, we did not assess alternative sources of income (e.g. student loans, parental support) and therefore unable to establish if the sample is truly economically disadvantaged. (3) The focus group questions were less personalized (referring to young men in general rather than themselves) to elicit openness in responses but this meant we were unable to determine if these motivators and barriers were experienced by the participants themselves or were more a perception of the experiences of young men in general. This may explain why few differences were identified by weight status categories. (4) Although responses were stratified by BMI, we did not separate by young male subgroups (e.g. unemployed, students, fathers etc) and future research may consider this to inform development of programs aiming to individually tailor content to specific subgroups of young men.

## Conclusions

Young men are classed as a ‘hard to reach group’ and are difficult to engage in preventive health interventions [13, 52]. Unique motivators and barriers for young men in the current research, which differ to studies in different ages and sex population samples, emphasise the importance of consulting young men to inform development of healthy lifestyle programs that aim to target them for the promotion of healthy eating and physical activity. These findings suggest that future interventions targeting physical activity and healthy eating for young men should include strategies to promote benefits relating to physical health, mental health, physical appearance, sport/ performance, and social influences of physical activity and healthy eating to facilitate engagement. Addressing key intrinsic, lifestyle, cognitive-emotional, logistic and social barriers to healthy eating and physical activity may help engage this target group. Future research is needed to identify the most effective ways to address these motivators and barriers within interventions.
